# Maintenance of contractile force of the hind limb muscles by the somato-lumbar sympathetic reflexes

**DOI:** 10.1186/s12576-021-00799-w

**Published:** 2021-05-21

**Authors:** Harumi Hotta, Kaori Iimura, Nobuhiro Watanabe, Kazuhiro Shigemoto

**Affiliations:** 1 Department of Autonomic Neuroscience, Tokyo Metropolitan Institute of Gerontology, 35-2 Sakaecho, Itabashi-ku, Tokyo, 173-0015 Japan; 2 Department of Geriatric Medicine, Tokyo Metropolitan Institute of Gerontology, Tokyo, 173-0015 Japan

**Keywords:** Lumbar sympathetic trunk, Skeletal muscles, Contractile force, Spinal cord, Dorsal roots, Somato-sympathetic reflex

## Abstract

This study aimed to clarify whether the reflex excitation of muscle sympathetic nerves induced by contractions of the skeletal muscles modulates their contractility. In anesthetized rats, isometric tetanic contractions of the triceps surae muscles were induced by electrical stimulation of the intact tibial nerve before and after transection of the lumbar sympathetic trunk (LST), spinal cord, or dorsal roots. The amplitude of the tetanic force (TF) was reduced by approximately 10% at 20 min after transection of the LST, spinal cord, or dorsal roots. The recorded postganglionic sympathetic nerve activity from the lumbar gray ramus revealed that both spinal and supraspinal reflexes were induced in response to the contractions. Repetitive electrical stimulation of the cut peripheral end of the LST increased the TF amplitude. Our results indicated that the spinal and supraspinal somato-sympathetic nerve reflexes induced by contractions of the skeletal muscles contribute to the maintenance of their own contractile force.

**Supplementary Information:**

The online version contains supplementary material available at 10.1186/s12576-021-00799-w.

## Introduction

During exercise, the autonomic nervous system regulates various autonomic functions, such as circulation, respiration, and body temperature, to maintain an internal environment that is suitable for exercise. In humans, muscle sympathetic nerves provide excitation during voluntary exercise, which is thought to be the result of central command from the brain and a reflex initiated in the contracting muscles [1–3]. In animals in which the effects of central command have been excluded by decerebration or anesthesia, the contraction of the triceps surae muscles by motor nerve stimulation elicits reflex activity in sympathetic efferents in the muscle nerve [4], lumbar sympathetic trunk (LST) [5], and renal nerve [5, 6]. Group III and IV somatic afferent nerves that are responsive to skeletal muscle contraction are involved as the afferent limb of the reflex arc, whereas the brainstem and spinal cord function as the reflex center [6–10]. However, it is not clear how the reflex activation of these sympathetic nerves induced by skeletal muscle contraction affects the function of the contracting muscles.

The unmyelinated postganglionic sympathetic efferent fibers, which represent 40% of all nerve fibers in the muscle nerve [9], have been shown histologically to distribute not only to vascular smooth muscle cells, but also to skeletal muscle fibers in the skeletal musculature [11, 12]. Clinically, sympathomimetics potentiate skeletal muscle strength [13–15]. Recent studies of mouse muscles showed that the size of neuromuscular junctions was decreased [16] and the contractile force generated by plantar nerve stimulation in in vitro nerve–muscle preparation was reduced [17] 1–2 weeks after sympathectomy, which suggests that sympathetic nerves are important for maintaining skeletal muscle functions.

Based on the two research trends described above, we hypothesized that the somato-muscle sympathetic reflex associated with contractions of skeletal muscles helps maintain their contractile force. To test this hypothesis, we first compared the contractile force of the triceps surae muscles induced by motor nerve stimulation before and after acute disconnection of the sympathetic efferent path in anesthetized rats. The results showed that the contractile force was decreased after the transection, indicating that sympathetic nerve activity may have an inotropic effect on skeletal muscles. Therefore, we then investigated the origin of the sympathetic nerve activity by disconnecting the afferent path or central path of the reflex, and concurrently recording the sympathetic efferent nerve activity going into the hind limbs. Last, we examined whether the contractile force increases during electrical stimulation of the sympathetic efferent nerve.

## Methods

Experiments were performed on 22 male Fischer rats (age, 4–10 months; weight, 310–395 g). The rats were used in five different experiments, as summarized in Table 1. To evaluate the effects of acute transection of the efferent, central, or afferent path of the reflex arc on the contraction force of the triceps surae muscles, the LST (exp. 1), cervical spinal cord (exp. 2), or lumbar dorsal roots (exp. 3) were, respectively, cut. Furthermore, we recorded (exp. 4) and stimulated (exp. 5) the sympathetic efferent nerve to the hind limb. These experiments were conducted in accordance with the Guidelines for Proper Conduct of Animal Experiments, which was established by the Science Council of Japan in 2006 and approved by the Animal Care and Use Committee of the Tokyo Metropolitan Institute of Gerontology.

**Table 1:** Summary of the experimental protocols

Recording	Condition		*n*
Contraction force	Exp. 1. before and after LST transection	Exp. 5. stim. cut LST	8
	Exp. 2. before and after spinal transection	–	5
	Exp. 3. before and after dorsal root transection	–	5
Sympathetic nerve activity	Exp. 4. before and after spinal transection	–	4

LST: lumbar sympathetic trunk; stim.: stimulation; exp.: experiment

### Basic preparation

All experimental procedures and observations were performed under urethane anesthesia. Basic preparations, including anesthesia and artificial respiration, and recordings of blood pressure and heart rate were essentially identical to those described in a previous study [18]. Animals were anesthetized with urethane (initial dose, 1.1–1.2 g/kg of body weight, administered subcutaneously (s.c.)) after initial inhalation of 3–5% sevoflurane at least for 3 min. Respiration was maintained using a ventilator (SN-480-7, Shinano, Tokyo, Japan) via a tracheal cannula, and end-tidal CO_2_ level was monitored using a gas analyzer (Capnostream 20P, Oridion Medical, Jerusalem, Israel) and maintained at approximately 3.5%. The rectal body temperature was kept at 37–38 °C using an automatically regulated heating pad and lamp (ATB-1100, Nihon Kohden, Tokyo, Japan). The jugular vein was catheterized for the intravenous (i.v.) administration of supplemental anesthetics and other drugs. Finally, the common carotid artery was catheterized to record arterial blood pressure and heart rate with a pressure transducer (TP-200T, Nihon Kohden) connected to a preamplifier (PB-100, Unique Medical, Tokyo, Japan).

The depth of anesthesia was routinely judged by observing the animal’s motion, respiration, blood pressure, and heart rate. If these conditions became unstable, 0.1–1.0% sevoflurane was administered via inhalation during surgery, or additional doses of urethane (0.06–0.14 g/kg, i.v.) were administered during the experiment. An electrolyte solution (Lactec D solution, Otsuka Pharmaceutical, Tokushima, Japan) was administered i.v. every hour to prevent dehydration. Rats were euthanized by injecting an overdose of pentobarbital at the end of each experiment.

### Recording of isometric contraction force of hind limb muscles

The left tibial nerve was isolated at approximately 25 mm centrally from the knee joint, and two flexible silver wire electrodes were attached to the intact tibial nerve, for stimulation. To avoid the confounding effect of antagonistic muscle contraction, a portion of the common peroneal nerve (approximately 10 mm in length) located near the electrodes was removed in advance. A sheet of parafilm (4 mm square) was placed under the electrodes to prevent the diffusion of the current to the underlying tissues. Subsequently, the electrodes and the nerve segment that was located between them were shielded with fixative silicon glue.

Animals were placed on either a supine (exps. 1, 4, and 5) or prone (exps. 2 and 3) position. The left hind limb was fixed to a metal frame in a plantar-flexed position, and the left calcaneal tendon was exposed via a partial skin incision and was tied with a thread connecting to a force displacement transducer (Ultra Small-Capacity Load Cell, LVS-20GA, Kyowa Electronics Instruments, Tokyo, Japan, 200 mN capacity) combined with a preamplifier (AP-610J, Nihon Kohden). A small weight (< 1 g) was used to make the thread horizontal without sagging. Repeatability and linearity were checked using calibration weights within the measurement ranges.

**Fig. 1:**
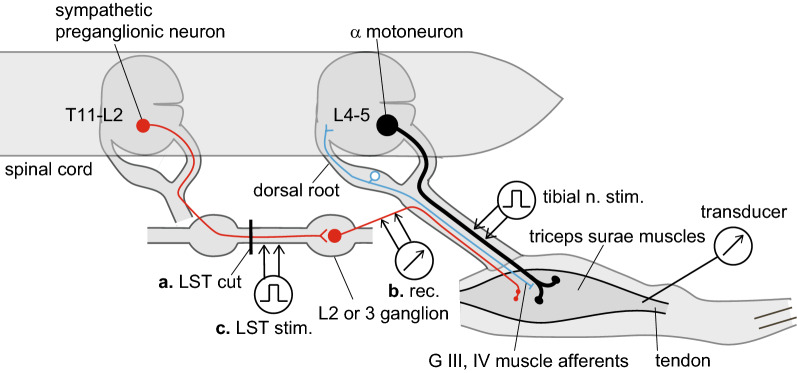
Schematic representation of electrode placement when transecting (**a**), recording (**b**), or stimulating (**c**) lumbar sympathetic nerve fibers while stimulating the tibial nerve. LST, lumbar sympathetic trunk

The left tibial nerve was stimulated electrically with a stimulator (SEN-7203, isolator; SS-202J, Nihon Kohden) using rectangular electrical pulses with a duration of 0.2 ms. At the beginning of each experiment, we tested single 0.2-ms pulse stimulation with various current intensities delivered to the left tibial nerve, to determine the threshold intensity (T) that evoked a visible twitch of the triceps surae muscle under a microscope. Current intensity was set at twice the motor threshold (2T), to stimulate motor fibers without directly activating small-diameter fibers, such as sympathetic postganglionic efferent and group III and IV afferent nerve fibers (Fig. 1).

**Fig. 2:**
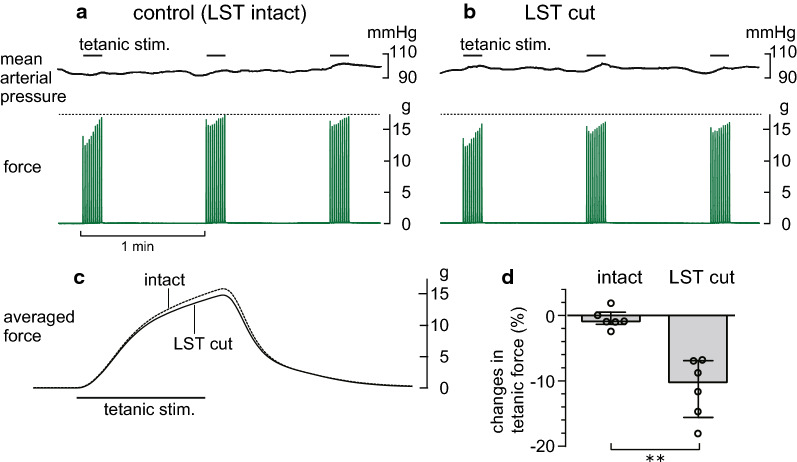
Effect of lumbar sympathetic trunk transection on the tetanic force of the triceps surae muscles. **a**, **b** Typical responses of the tetanic force of the triceps surae muscles (lower traces) and mean arterial pressure (upper traces) induced by tetanic stimulation of the intact tibial nerve before (**a**) and 20 min after (**b**) lumbar sympathetic trunk (LST) transection. Each upper horizontal bar indicates the tetanic stimulation (0.2 ms, 2T, 10 pulses at 100 Hz) repeated 10 times at 1 Hz. **c** Averaged waveform of the 30 tetanic contractions before (dotted line) and after (solid line) LST transection. **d** Summary (*n* = 6 from six rats) of the changes in the amplitude of the tetanic force, expressed as a percentage of the mean of two controls obtained before LST transection. Each column, vertical bar, or circle indicates the median, interquartile range, and individual data. **p < 0.01 (Mann–Whitney *U* test)

A tetanic contraction of the left triceps surae muscles was produced by train stimuli consisting of 10 pulses at 100 Hz, according to previous studies [19, 20]. To avoid fatigue, 10 tetanic contractions at 1 Hz were repeated 3 times at an interval of 1 min (Fig. 2a). We averaged the waveform of the 30 tetanic contractions and treated them as one set of tetanic contractions.

### Transection of the LST

The abdomen was opened to expose the LST. Usually, the renal artery on the left side lies near the L2 sympathetic ganglion, and postganglionic sympathetic neurons to the triceps surae muscles reportedly exist mainly in the L2–4 sympathetic ganglia in rats [21]; moreover, their preganglionic neurons are present in the spinal cord at a more rostral location, i.e., the T11–L2 level [22]. Therefore, we transected the unilateral LST close to the left renal artery, i.e., either rostral or caudal to the L2 ganglion (Fig. 1, a. LST cut), to eliminate the majority of sympathetic efferent nerve activity going to the ipsilateral triceps surae muscles. In preparation for subsequent cutting, a ligature was loosely attached to the LST. After obtaining control sets of tetanic contractions using the intact LST, the LST was transected (exp. 1).

### Transection of the spinal cord

The dorsal neck was incised to expose the spinal cord at the first cervical level. After obtaining control sets of responses with intact central nervous system, the spinal cord was transected (exps. 2 and 4). Systolic blood pressure was maintained above 70 mmHg via the intravenous administration of 4% Ficoll PM70 solution.

### Transection of the dorsal root innervating the hind limbs

Laminectomy was performed at the L3 vertebrae to expose the dorsal roots below L3, which includes afferent nerve fibers from the triceps surae muscles [21]. After obtaining control responses using intact dorsal roots, the left L3–6 dorsal roots were transected (exp. 3). The level of transection of the dorsal roots was confirmed postmortem.

### Recordings of the activity of the postganglionic sympathetic nerve innervating the hind limbs

Mass nerve activity was recorded from the gray ramus derived from lumbar sympathetic ganglia (Fig. 1, b. rec.), which is the specific pathway of the sympathetic postganglionic fibers innervating the hind limbs (exp. 4). A nerve bundle from the caudal portion of the L2 or L3 paravertebral sympathetic ganglia on the left side was cut as close as possible to the spinal nerve and covered with liquid paraffin. Efferent discharge activity was recorded from the central cut end of the nerve, which was placed on a bipolar platinum–iridium wire electrode and amplified using a preamplifier (MEG-6100, Nihon Kohden). Discharge activity was continuously monitored with an oscilloscope and speaker, to prevent contamination with any recording artifacts that might occur. The band of 200–1000 Hz was extracted and rectified using the Spike2 software (Cambridge Electronic Design, Cambridge, UK). The reflex responses elicited by electrical stimulation of the left tibial nerve were averaged (60 trials) using the software. The reflex potentials were averaged every 5 ms, normalized as a percentage of prestimulus values, and summarized in the intact CNS and spinalized conditions. At the end of the experiments, we injected a ganglionic blocker, i.e., hexamethonium (20 mg/kg, i.v.), and observed the disappearance of the nerve activity, thereby confirming that the discharge was a postganglionic efferent discharge.

### Electrical stimulation of the LST

To stimulate the sympathetic nerve fibers that innervate the hind limbs, the LST was cut unilaterally on the left side, and the cut peripheral end of the LST was placed on a bipolar platinum–iridium wire stimulation electrode (Fig. 1, c. LST stim.). The nerve was covered with warm liquid paraffin. Based on stimulus parameters that are known to be supramaximal for all nerve fibers in the LST, the stimulus intensity and pulse duration were maintained at 8–10 V and 0.5 ms, respectively [23]. Three to five different frequencies ranging from 1 to 50 Hz were tested in each rat. Sham stimulation was also tested in four animals. The order of stimulus frequency was randomized. To reduce the number of animals used in our experiments, we performed this experiment (exp. 5) right after completing experiment 1. We tested LST stimulation more than 1 h after LST transection.

### Analysis and statistics

All obtained analog signals were digitized (Micro 1401, Cambridge Electronic Design) and displayed on a computer monitor for online and offline analysis using the Spike2 software. The force signal was sampled at 2 kHz and smoothed at a time constant of 5 ms. The values obtained after transection (LST, spinal cord, or dorsal root) or stimulation (LST) were compared with the values obtained before the transection or stimulation (pretest–posttest design). Sets of 30 tetanic contractions were repeated at 10-min intervals and, if the amplitude of consecutive sets was stable in the control condition, the LST, spinal cord, or dorsal roots were transected. After transection, the sets of tetanic contractions were repeated at an interval of 10 min. The peak amplitude of the averaged waveform of 30 tetanic contractions was evaluated as the tetanic force (TF). Percent changes in TF at 20 min after transection of the LST, spinal cord, and dorsal roots, respectively, were compared to those of the corresponding intact control (just before the transection) using the Mann–Whitney *U* test. The Wilcoxon signed-rank test was used to compare absolute values of TF or mean arterial pressure between the prestimulus control and the LST stimulation. Values are expressed as medians and interquartile ranges, unless otherwise stated. Statistical difference of time course of reflex potentials between the intact CNS and spinalized conditions was analyzed by two-way repeated-measures analysis of variance (ANOVA) (Prism 6, GraphPad Software, CA, USA). Statistical significance was set at *p* < 0.05.

## Results

### Effects of the transection of the unilateral LST on the TF of the triceps surae muscles (exp. 1)

The effects of the transection of the unilateral LST on the tetanic contractions of the triceps surae muscles induced by tibial nerve stimulation were examined in eight rats. Figure 2a and b provides an example of the recording of the tetanic contractions in the control condition, with an intact LST (a), and of those recorded 20 min after LST transection (b) on the left side, which was ipsilateral to the contracting muscles. The maximum force in the case of the intact LST is indicated by the horizontal dashed line, which was higher than that of the maximum force recorded after LST transection. Conversely, the systemic mean arterial pressure recorded before and during the tetanic tibial nerve stimulation was not different between the two conditions (control with intact LST and after unilateral LST transection), as shown in the upper traces presented in Fig. 2a and b. The changes in arterial pressure associated with tetanic contractions varied in individual animals, i.e., increase, decrease, or unchanged, but were consistent between the two conditions. Furthermore, for precise quantification, the TF waveforms were averaged across all 30 repetitions in each condition and were superimposed to compare the conditions, as shown in Fig. 2c. After LST transection, the peak time of the TF generation remained unchanged, whereas the peak amplitude was slightly smaller than that of the control TF.

A similar result was observed in all six rats that underwent transection of the LST ipsilateral to the contracting muscles. The attenuation was observed as early as 10 min after the transection and was then further reduced 20 min later, and it subsequently remained at a low level for 30 min. The graph depicted in Fig. 2d summarizes the changes in TF amplitude from immediately before to 20 min after LST transection, which are expressed as a percentage of the averaged two sets of contractions recorded before LST transection in the six rats. The force was significantly reduced by a median value of 10.2% (interquartile range, 6.9–15.6%) after acute transection of the LST ipsilateral to the contracting muscles (*p* < 0.01). The changes in TF occurred only when the LST ipsilateral to the contracting muscles was transected. No change in TF was observed when the LST contralateral to the contracting muscles was transected in the remaining two rats.

The tetanic force amplitude in the control condition varied from 0.9 to 24 g in the individual rats described above. However, regardless of the absolute value of the original force amplitude, the force amplitude was decreased following transection of the ipsilateral LST by approximately 10% of the original force. The same effect was also observed in an additional rat with a basal force of 160 g (see Additional file 1 for modification of the method for this case); therefore, there was a significant correlation between basal force and delta changes following transection of the ipsilateral LST (Additional file 1).

### Effects of spinalization and/or dorsal root transection on the TF of the triceps surae muscles (exps. 2 and 3)

**Fig. 3:**
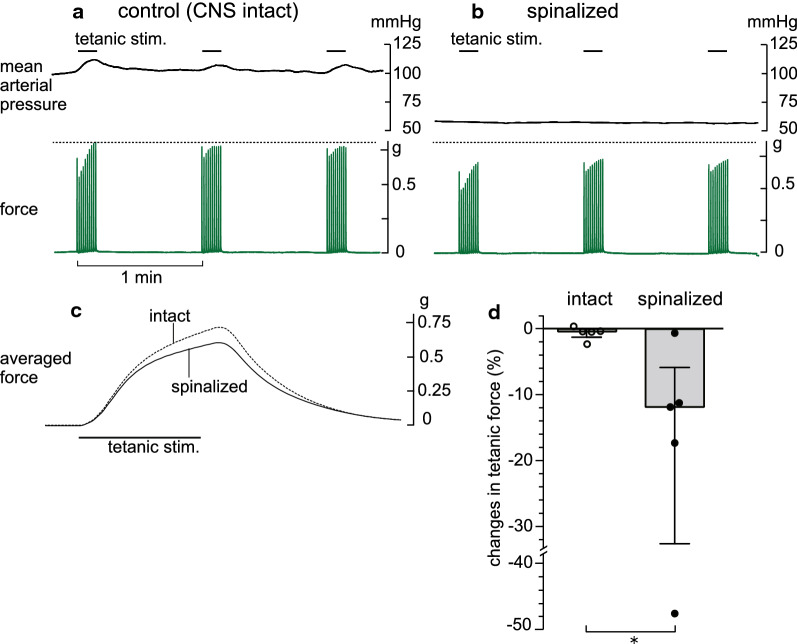
Effect of spinal cord transection on the tetanic force of the triceps surae muscles. **a**, **b** Typical responses of the tetanic force of the triceps surae muscles and mean arterial pressure induced by tetanic stimulation of the intact tibial nerve before (**a**) and 20 min after (**b**) spinal cord transection. **c** Averaged waveform of the 30 tetanic contractions before (dotted line) and after (solid line) spinal cord transection. **d** Summary (*n* = 5 from five rats) of the changes in the amplitude of the tetanic force, expressed as a percentage of the mean of two controls obtained before spinal cord transection. Each column, vertical bar, or circle indicates the median, interquartile range, and individual data. *p < 0.05 (Mann–Whitney *U* test)

The results of acute LST transection reported above suggest that the sympathetic efferent nerve activity conveyed in the LST potentiates the generation of TF in the triceps surae muscles. To investigate the possibility that the sympathetic nerve activity that modulates the TF is derived from a descending input from the brain, the spinal cord was severed at the cervical level in five rats. Figure 3a and b shows an example recording of the tetanic contractions and mean arterial pressure in the control condition, with an intact CNS (a), and those recorded 20 min after spinal cord transection (b). The basal level of systemic mean arterial pressure was decreased by acute spinalization, as expected. The maximum force recorded under the intact CNS was decreased after spinalization. Although the peak time of the averaged TF remained unchanged, the peak amplitude after spinalization was smaller than that of the control TF (Fig. 3c). Similar results were observed in four of the five rats tested. In the remaining rat, TF was not considerably affected after spinalization despite a consistent decrease in blood pressure. In summary, TF was significantly reduced by 11.8% 20 min after spinalization compared with the control level (*p* < 0.05), which was detected before spinalization (Fig. 3d). However, the extent of the reduction varied from 0.6% (minimum) to 48% (maximum), depending on the individual animals.

**Fig. 4:**
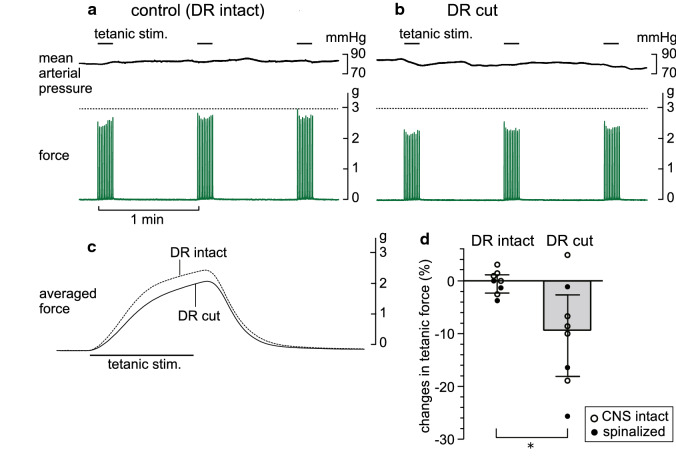
Effect of dorsal root transection on the tetanic force of the triceps surae muscles. **a**, **b** Typical responses of the tetanic force of the triceps surae muscles and mean arterial pressure induced by tetanic stimulation of the intact tibial nerve before (**a**) and 20 min after (**b**) dorsal root transection in a rat with an intact CNS. **c** Averaged waveform of the 30 tetanic contractions before (dotted line) and after (solid line) dorsal root transection in the same rat. **d** Summary (*n* = 8 from eight rats) of the changes in the amplitude of the tetanic force, expressed as a percentage of the mean of two controls obtained before dorsal root transection. Each column, vertical bar, or circle shows the median, interquartile range, and individual data. Open circles: data from rats with an intact CNS; closed circles: data from rats with spinal cord transection. *p < 0.05 (Mann–Whitney *U* test)

To examine whether the sympathetic nerve activity conveyed by the LST that affects the TF reflects activation by afferent information from peripheral tissues in response to the contraction of the triceps surae muscles, the left ipsilateral L3–6 spinal dorsal roots, through which afferent fibers from the hind limbs pass, were cut. For this experiment, we used five rats with an intact CNS and three of the five spinalized rats described above. In these rats, the effect of dorsal root transection was tested at more than 1 h after spinalization. Figure 4a and b shows an example recording of the tetanic contractions and mean arterial pressure of the control condition before (a) and of 20 min after (b) dorsal root transection in a rat with an intact CNS. The basal level of systemic mean arterial pressure was unchanged between the intact and cut dorsal roots (Fig. 4a, b). The maximum force recorded under the intact dorsal roots was decreased after transection. The peak time of the averaged TF remained unchanged, whereas the peak amplitude after dorsal root transection was smaller than that of the control TF (Fig. 4c). Similar results were observed in the five rats tested with the intact CNS condition, except for one rat in which TF increased after dorsal root transection. The effect of spinal dorsal root transection was similarly observed in spinalized rats. In both cases, the degree of changes varied among individuals, ranging from 4.9% increase to 19.0% decrease in rats with an intact CNS (open circles in Fig. 4d), and from 1.1% to 25.7% decrease in those with acute spinalization (closed circles in Fig. 4d). Altogether, TF was significantly reduced by 9.3% (*p* < 0.05, Fig. 4d).

### Recording of postganglionic sympathetic efferent nerve activity innervating the hind limbs (exp. 4)

The results described above suggest that both supraspinal and spinal somato-sympathetic reflexes are at the origin of the sympathetic efferent nerve activity that potentiates the contractile force of the triceps surae muscles. Therefore, ipsilateral postganglionic sympathetic efferent nerve activity to the hind limbs was recorded from the gray ramus of the left L2 or L3 lumbar paravertebral ganglia, to confirm whether reflex activities are elicited in response to contractions. The reflex responses associated with isometric contractions of the triceps surae muscles were analyzed in both the intact CNS condition and the acute spinalization condition in four rats.

**Fig. 5:**
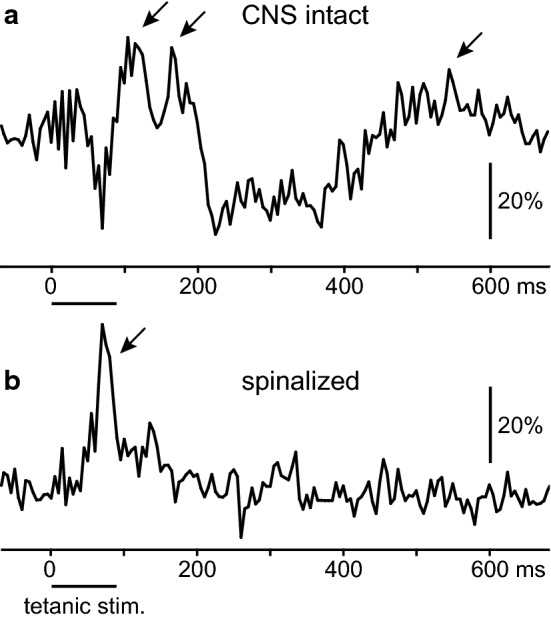
Reflex potentials in postganglionic sympathetic nerves evoked by tetanic stimulation of the tibial nerve. The reflex responses in sympathetic nerves averaged for 60 trials were summarized in the intact CNS (**a**) and spinalized (**b**) conditions (*n* = 4 from four rats). The lower horizontal bars indicate the tetanic stimulation (0.2 ms, 2T, 10 pulses at 100 Hz). The arrows indicate excitatory potentials. Vertical bars indicate percent changes relative to the prestimulus values

In the intact CNS condition, a distinct rhythm of sympathetic nerve activity synchronized to the heartbeat was observed. The rhythm changed with the initiation of tetanic contractions of the triceps surae muscles. Therefore, we averaged the nerve activity triggered by the onset of tetanic stimuli of the tibial nerve. As shown in Fig. 5a, the stimulation elicited sympathetic reflex responses that consisted of two early excitations, post-excitatory inhibition, and late excitation. The latencies of the two early excitations were 83 ms (range 78–94 ms) and 146 ms (145–150 ms), respectively, and the latency of the late excitation was 419 ms (370–435 ms) in the four rats.

In all four rats, the spinal cord was transected at the first cervical level and reflex responses were recorded 0.5–2 h after spinal transection. In the spinalized condition, the postganglionic sympathetic nerve activity lost synchronization with the heartbeat, and spontaneous nerve activity was markedly reduced, as reported previously for the rat adrenal nerve [24] and the human muscle sympathetic nerve [25]. However, excitatory reflex potentials were clearly observed in association with tetanic stimulation of the intact tibial nerve. The latency of the reflex potential was 49 ms (range, 40–55 ms) (Fig. 5b), which was shorter than that recorded in the intact CNS condition in all tested cases. According to the two-way ANOVA, the main effect of the condition was not significant, but time (*p* < 0.01) and interaction (*p* < 0.01) were significant, indicating that there was a significant difference in the time course of reflex potentials between the two conditions.

At the end of experiments performed after spinalization, the muscles were immobilized via the administration of a muscle relaxant (vecuronium bromide, 2 mg/kg, i.v.). With the loss of muscle contraction, the reflex potentials detected after tetanic tibial stimulation at the current intensity of 2T were almost abolished (Additional file 2a). This result indicates that the potentials were produced secondarily by muscle contractions, rather than being caused by direct electrical stimulation of the tibial nerve afferents. In this condition, the increase in the stimulus strength of the tibial nerve to a supramaximal intensity (0.5 ms, 8–20 V), which excites all nerve fibers, including group III and IV afferent fibers, as reported previously [26], elicited reflex potentials (tested in three of the four rats, Additional file 2b). The latency was 43 ms (range, 40–45 ms), which was similar to that induced secondarily by muscle contractions, as described above (Fig. 5b).

### Effect of LST stimulation on the TF of the triceps surae muscles (exp. 5)

**Fig. 6:**
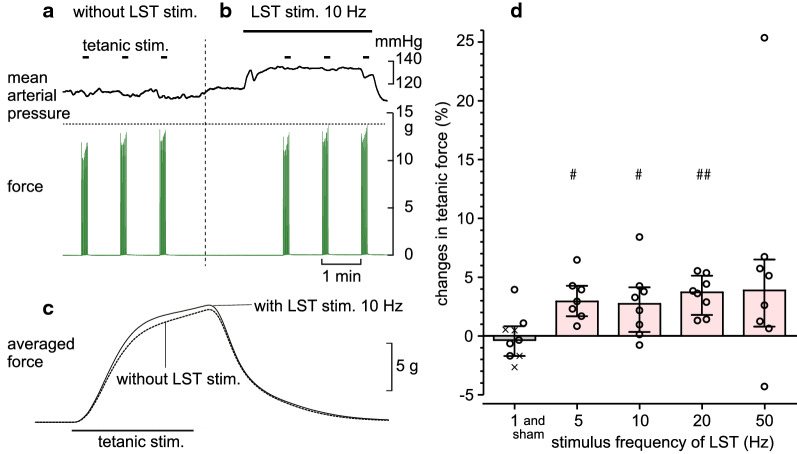
Effect of lumbar sympathetic trunk stimulation on the tetanic force of the triceps surae muscles. **a**, **b **Typical responses of the tetanic force of the triceps surae muscles and mean arterial pressure induced by tetanic stimulation of the intact tibial nerve before (**a**) and during (**b**) stimulation (10 Hz) of the cut peripheral end of the LST. **c** Averaged waveform of the 30 tetanic contractions without (dotted line) and with (solid line) LST stimulation. **d** Summary (*n* = 7–9 from eight rats) of the changes in the amplitude of the tetanic force, expressed as a percentage of the prestimulus control level. Each column or vertical bar indicates the median and interquartile range. Each circle indicates individual data for stimulation. Data of sham stimulation are shown as x. Horizontal axis: frequency of LST stimulation. Vertical axis: changes in the peak amplitude of the tetanic force, expressed as a percentage of the control just before LST stimulation. #*p* < 0.05, ##*p* < 0.01 (Wilcoxon signed-rank test; compared with the peristimulus control using absolute values of tetanic force)

To confirm that the efferent activity of the sympathetic nerve fibers contained in the LST enhances the TF of triceps surae muscles, we stimulated the cut peripheral end of the LST in eight rats. In these experiments, we started LST stimulation 1 min before the onset of 30 tetanic contractions, and compared the averaged force of the 30 tetanic contractions of the prestimulus control with that of the LST stimulation (Fig. 6).

Figure 6a–c depicts a representative recording of TF and mean arterial pressure after repetitive stimulation of the LST ipsilateral to the contracting muscles at a frequency of 10 Hz and a supramaximal intensity of 10 V. The systemic mean arterial pressure increased during LST stimulation (Fig. 6a, b), as reported previously [23]. The TF induced by tibial nerve stimulation also increased during LST stimulation (Fig. 6b, c). The effects of LST stimulation were dependent on stimulus frequency. The TF was minimally affected by LST stimulation at 1 Hz, whereas it was enhanced at frequencies above 5 Hz. Moreover, the TF was significantly increased during LST stimulation at frequencies between 5 and 20 Hz compared with the corresponding prestimulation control force. In addition, stimulation at 50 Hz increased the TF in most cases; however, this result was not significant because of a large variability. Figure 6d summarizes the changes in TF during LST stimulation at various frequencies, which are expressed as a percentage of the prestimulus control force. Stimulation at 5, 10, 20, and 50 Hz increased the TF by 2.9% (1.7%–4.3%, n = 7, *p* < 0.05), 2.7% (0.3%–4.1%, n = 8, *p* < 0.05), 3.7% (1.8%–5.1%, n = 8, *p* < 0.01), and 3.9% (0.7%–6.5%, n = 8, *p* = 0.078), respectively. Concomitantly, the mean arterial pressure increased by 9.1% (3.5%–15.3%, *p* < 0.05), 18.7% (17.4%–22.6%, *p* < 0.01), 18.2% (14.7%–24.4%, *p* < 0.01), 17.7% (14.4%–29.0%, *p* < 0.01), respectively.

## Discussion

**Fig. 7:**
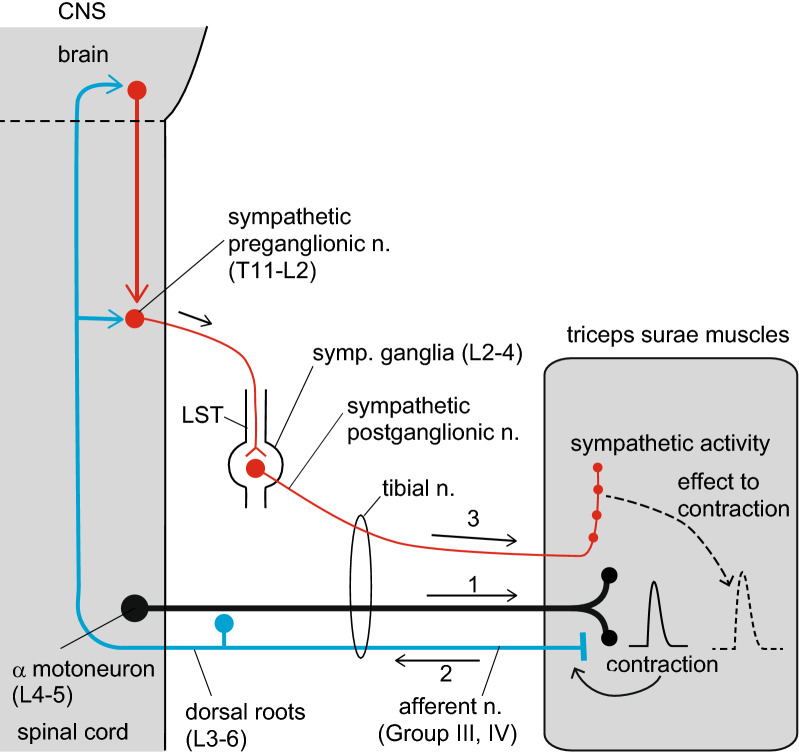
Schematic diagram showing the sympathetic excitation and increase in muscle force afforded by muscle contraction. When the alpha motor neurons innervating the triceps surae muscles where cell bodies are localized in the L4–5 spinal cord and their axons included in the tibial nerve are excited and the triceps surae muscles contract (route 1), the group III and IV afferents of the muscles are excited (route 2). The afferent information is transmitted to the central nervous system via the L3–6 dorsal roots, and then reflexively excites the sympathetic preganglionic neurons in the spinal cord (T11–L2), which are connected through LST to the postganglionic neurons in the L2–4 sympathetic ganglia. Subsequently, the sympathetic postganglionic efferent nerve activity reaches the triceps surae muscles (route 3) and increases their contractile force

In this study, we found that the force of motor nerve-induced tetanic contractions was decreased by the elimination of sympathetic nerve activity, which was achieved by transecting the LST, whereas it increased by its activation via the stimulation of the cut LST. The LST is a selective pathway of sympathetic nerve fibers and does not include somatic motor axons [27], so that acute surgical transection of the LST selectively eliminates lumbar sympathetic nerve activity going to the lower body without damaging the somatic motor axons. Therefore, our findings indicate that the sympathetic nerve activity in the LST has a positive inotropic effect on hind limb muscles. Furthermore, we showed that sympathetic nerve activity originated in both the descending and segmental synaptic input to the sympathetic preganglionic neurons in the spinal cord, because transection of either the spinal cord or dorsal roots decreased the TF to a similar extent as did LST transection. The reflex potentials observed in the hind limb postganglionic sympathetic nerve discharges induced by tetanic contractions supported this evidence. Therefore, we concluded that the sympathetic reflexes evoked via the brain and spinal cord and triggered by afferent information from contracting muscles (somato-autonomic reflexes) are involved in the potentiation of the TF. Figure 7 summarizes the results and conclusions of the present study.

### Sympathetic efferent mechanisms

Our results showed that the TF in the triceps surae muscles was reduced by approximately 10% after acute transection of the LST. This result is consistent with a recent study that reported a lower TF (by approximately 16%) in a plantar nerves–lumbricalis muscle preparation dissected from mice 7 days after the removal of L2 and L3 lumbar sympathetic ganglia than that from control mice with intact sympathetic ganglia [17]. However, in that report, the cross-sectional area of the muscle was decreased by approximately 20% because of chronic sympathectomy; therefore, the decrease in TF may be attributed to muscular atrophy. To the best of our knowledge, the present results showed for the first time that the sympathetic nerve activity in the LST ipsilateral to the contracting muscles plays a role in the maintenance of their contractile force in vivo in normal physiological conditions without muscle atrophy.

The stimulation of the peripheral cut end of the LST at frequencies of 5–20 Hz increased the contraction force in rat triceps surae muscles by 3–4%. Similar results were reported previously, e.g., the stimulation of the cervical sympathetic trunk at 10 Hz induced an increase of approximately 10% in the maximal twitch force in directly stimulated rabbit mandibular muscles [28]; and LST stimulation at 5 Hz increased the TF of rat gastrocnemius–plantaris muscles in parallel with increases in arterial pressure [19]. In these studies, similar effects were observed by local arterial injection of adrenoceptor agonists. Our results showed that changes in the TF did not always occur in parallel with changes in arterial pressure; both parameters increased and decreased after LST stimulation and spinal cord transection, respectively; however, the TF decreased without changes in arterial pressure after transection of the LST or dorsal roots. The changes observed in the TF in all four different conditions (LST transection, LST stimulation, spinalization, and dorsal root transection) can be explained by variations in muscle sympathetic nerve activity, but not by variations in arterial pressure.

### Central nervous system mechanisms

It is a well-known fact that in humans and animals with chronic spinal cord transection, the TF is reduced in the hind limb muscles. For example, 1 month after complete spinal cord transection (at the T9–10 level), the TF was decreased in most motor units of the medial gastrocnemius muscle of the rat [29]. In this chronic condition, the muscles were atrophied to 60% of the spinal intact controls. However, we were unable to find any studies that examined the effect of acute spinal cord transection on skeletal muscle contractility. We showed for the first time that the reduction of the TF started as early as 20 min after acute spinalization. Therefore, reduction of the TF after spinal cord injury can be initiated before muscle atrophy, possibly because of the decrease of sympathetic efferent nerve activity innervating the hind limbs, as observed in the present nerve-recording study. It was also reported in humans that spontaneous muscle sympathetic nerve activity was much lower in patients with spinal cord lesions than in normal subjects [25].

The analysis of reflex potentials in the autonomic efferent nerves evoked by somatic afferent nerve stimulation is useful to examine the central reflex pathways of somato-autonomic reflexes [8]. In our recordings, the sympathetic activities to the skin may have contaminated the results; however, those to muscles seem to be predominant, based on clear heart-rate-related rhythmicity [30] and responsiveness to muscle contractions. These are features of muscle sympathetic nerves and are different from those of skin sympathetic nerves [4, 31, 32]. We showed that various reflex components were induced in the postganglionic sympathetic efferents to the hind limbs via contraction of the ipsilateral triceps surae muscles, in both the intact CNS (latencies: 83 ms, 146 ms, and 419 ms) and spinalized (latency: 49 ms) conditions. The reflexes detected in the intact CNS condition seemed to be mediated mainly by supraspinal pathways, as they were nearly abolished by spinal transection at the cervical level. Another reflex component with a much shorter latency observed after spinalization, i.e., the spinal reflex component, seems to be suppressed, at least in part, by supraspinal effects. Such central mechanisms were similar to those reported in adrenal sympathetic nerve induced by the single-shock stimulation of a hind limb nerve [24].

The descending pathways of the muscle sympathetic nerve, which were mapped by the injection of a transneuronal tracer into the rat gastrocnemius muscles, include several medullary areas, e.g., the raphe nuclei, the rostral ventrolateral medulla, and the paraventricular nucleus of the hypothalamus [33]. The injection of glutamate into the ventral surface of the medulla activates the hind limb muscle sympathetic nerves in felines [34]. Further studies are needed to determine which of these brain regions are the centers of the descending regulation of the muscle sympathetic nerve that is involved in the regulation of muscle contractility, and whether they are distinct from that involved in the vascular regulation.

### Afferent mechanisms

Transection of the L3–6 dorsal roots of the spinal cord attenuated the TF in the hind limbs, indicating the presence of reflexes triggered by peripheral afferent information, most likely from the contracting hind limb muscles. The nerve activity of group III and IV somatic afferents in the dorsal roots, which affect autonomic nerve activity, was excited in response to the contraction of hind limb muscles [35–37]. Therefore, we predict that the excitation of group III and IV afferent fibers in contracting muscles reflexively modifies muscle sympathetic nerve activity. This assumption was supported by our recordings of postganglionic sympathetic discharges, which showed that the latency of the potential directly induced by the supramaximal electrical stimulation that excites group III and IV afferent fibers under immobilization (Additional file 2b) was similar to that indirectly induced via muscle contractions (Fig. 5b).

## Conclusion

In addition to the well-known functions of the autonomic nervous system in the regulation of circulation, respiration, and body temperature during exercise, a novel physiological mechanism was identified in which the activation of muscle sympathetic nerves during skeletal muscle contraction helps maintain muscle strength in the contracting muscles. The unmyelinated postganglionic sympathetic efferent fibers, which are a major component of the nerve fibers in muscle nerves, are activated by tetanic muscle contractions. It is our understanding that the physiological mechanism uncovered here is related to the improvement of muscle function normally induced by physical training. Conversely, a decline in this somato-sympathetic reflex mechanism may be a causative factor of muscle weakness in the elderly, in conditions of muscle disuse, and in several muscle diseases.

### Supplementary Information

**Additional file 1.** Correlation between basal force amplitudes and decrease in force amplitudes following transection of the ipsilateral LST. In the present method, the triceps surae muscles were minimally stretched, so that the basal force recorded (ranging between 0.9 g and 24 g in individual rats) was much less than that of the maximum force. This graph included an additional data, in which the nearly maximum force (160 g) was recorded by using modified methods as described below. The left hind limb was firmly fixed by using bone clamps (STS-A, Narishige, Tokyo, Japan), the triceps surae muscles were stretched with a weight of 50 g and connected to another model of transducer (LVS-1KA, Kyowa Electronics Instruments, 10 N capacity). Strength of correlation was analyzed by Pearson’s method.

**Additional file 2.** Lumbar sympathetic nerve activity in spinalized animals after the administration of muscle relaxants. **a**: The tetanic stimulation of the intact tibial nerve at 2 T, which caused tetanic contractions in the triceps surae muscles, scarcely induced any reflexive activity in the lumbar sympathetic nerve after muscle relaxation (*n* = 4 from four rats). **b**: Reflex discharges induced by single-pulse stimuli with a duration of 0.5 ms delivered every 3 s, with a supramaximal intensity (8–20 V) to excite group III and IV fibers of the tibial nerve after muscle relaxation (*n* = 3 from three rats). Vertical bars indicate percent changes relative to the prestimulus values.

## Data Availability

The datasets used and analyzed during the current study are available from the corresponding author on reasonable request.

## Abbreviations

LST: Lumbar sympathetic trunk
TF: Tetanic force
CNS: Central nervous system

## References

[1] Seals DR, Victor RG (1991). Regulation of muscle sympathetic nerve activity during exercise in humans. Exerc Sport Sci Rev.

[2] Boulton D, Taylor CE, Green S, Macefield VG (2018). The metaboreflex does not contribute to the increase in muscle sympathetic nerve activity to contracting muscle during static exercise in humans. J Physiol.

[3] Katayama K, Saito M (2019). Muscle sympathetic nerve activity during exercise. J Physiol Sci.

[4] Hill JM, Adreani CM, Kaufman MP (1996). Muscle reflex stimulates sympathetic postganglionic efferents innervating triceps surae muscles of cats. Am J Physiol.

[5] Koba S, Xing J, Sinoway LI, Li J (2007). Differential sympathetic outflow elicited by active muscle in rats. Am J Physiol Heart Circ Physiol.

[6] Victor RG, Rotto DM, Pryor SL, Kaufman MP (1989). Stimulation of renal sympathetic activity by static contraction: evidence for mechanoreceptor-induced reflexes from skeletal muscle. Circ Res.

[7] Sato A, Kaufman A, Koizumi K, Brooks CM (1969). Afferent nerve groups and sympathetic reflex pathways. Brain Res.

[8] Sato A, Sato Y, Schmidt RF (1997). The impact of somatosensory input on autonomic functions. Rev Physiol Biochem Pharmacol.

[9] Mitchell JH, Schmidt RF (1983) Cardiovascular reflex control by afferent fibers from skeletal muscle receptors. Supplement 8. Handbook of Physiology, The Cardiovascular System, Peripheral Circulation and Organ Blood Flow. 10.1002/cphy.cp020317

[10] Iwamoto GA, Waldrop TG, Kaufman MP, Botterman BR, Rybicki KJ, Mitchell JH (1985). Pressor reflex evoked by muscular contraction: contributions by neuraxis levels. J Appl Physiol.

[11] Barker D, Saito M (1981). Autonomic innervation of receptors and muscle fibres in cat skeletal muscle. Proc R Soc Lond B Biol Sci.

[12] Di Bona A, Vita V, Costantini I, Zaglia T (2020). Towards a clearer view of sympathetic innervation of cardiac and skeletal muscles. Prog Biophys Mol Biol.

[13] Lashley D, Palace J, Jayawant S, Robb S, Beeson D (2010). Ephedrine treatment in congenital myasthenic syndrome due to mutations in DOK7. Neurology.

[14] Ghazanfari N, Morsch M, Tse N, Reddel SW, Phillips WD (2014). Effects of the ß2-adrenoceptor agonist, albuterol, in a mouse model of anti-MuSK myasthenia gravis. PLoS ONE.

[15] Rodríguez Cruz PM, Cossins J, Cheung J, Maxwell S, Jayawant S, Herbst R, Waithe D, Kornev AP, Palace J, Beeson D (2020). Congenital myasthenic syndrome due to mutations in MUSK suggests that the level of MuSK phosphorylation is crucial for governing synaptic structure. Hum Mutat.

[16] Khan MM, Lustrino D, Silveira WA, Wild F, Straka T, Issop Y, O'Connor E, Cox D, Reischl M, Marquardt T, Labeit D, Labeit S, Benoit E, Molgó J, Lochmüller H, Witzemann V, Kettelhut IC, Navegantes LCC, Pozzan T, Rudolf R (2016). Sympathetic innervation controls homeostasis of neuromuscular junctions in health and disease. Proc Natl Acad Sci USA.

[17] Rodrigues ACZ, Messi ML, Wang ZM, Abba MC, Pereyra A, Birbrair A, Zhang T, O'Meara M, Kwan P, Lopez EIS, Willis MS, Mintz A, Files DC, Furdui C, Oppenheim RW, Delbono O (2019). The sympathetic nervous system regulates skeletal muscle motor innervation and acetylcholine receptor stability. Acta Physiol.

[18] Hotta H, Watanabe N (2015). Gentle mechanical skin stimulation inhibits micturition contractions via the spinal opioidergic system and by decreasing both ascending and descending transmissions of the micturition reflex in the spinal cord. PLoS ONE.

[19] Thomas GD, Hansen J, Victor RG (1994). Inhibition of α_2_-adrenergic vasoconstriction during contraction of glycolytic, not oxidative, rat hindlimb muscle. Am J Physiol.

[20] Schmoll M, Unger E, Sutherland H, Haller M, Bijak M, Lanmüller H, Jarvis JC (2017). In-situ measurements of tensile forces in the tibialis anterior tendon of the rat in concentric, isometric, and resisted co-contractions. Physiol Rep.

[21] Baron R, Jänig W, Kollmann W (1988). Sympathetic and afferent somata projecting in hindlimb nerves and the anatomical organization of the lumbar sympathetic nervous system of the rat. J Comp Neurol.

[22] Rotto-Percelay DM, Wheeler JG, Osorio FA, Platt KB, Loewy AD (1992). Transneuronal labeling of spinal interneurons and sympathetic preganglionic neurons after pseudorabies virus injections in the rat medial gastrocnemius muscle. Brain Res.

[23] Hotta H, Nishijo K, Sato A, Sato Y, Tanzawa S (1991). Stimulation of lumbar sympathetic trunk produces vasoconstriction of the vasa nervorum in the sciatic nerve via α-adrenergic receptors in rats. Neurosci Lett.

[24] Isa T, Kurosawa M, Sato A, Swenson RS (1985). Reflex responses evoked in the adrenal sympathetic nerve to electrical stimulation of somatic afferent nerves in the rat. Neurosci Res.

[25] Stjernberg L, Blumberg H, Wallin BG (1986). Sympathetic activity in man after spinal cord injury. Outflow to muscle below the lesion. Brain.

[26] Uchida S, Kagitani F, Hotta H (2008). Mechanism of the reflex inhibition of heart rate elicited by acupuncture-like stimulation in anesthetized rats. Auton Neurosci.

[27] Jänig W, McLachlan EM (1986). The sympathetic and sensory components of the caudal lumbar sympathetic trunk in the cat. J Comp Neurol.

[28] Grassi C, Deriu F, Roatta S, Santarelli R, Azzena GB, Passatore M (1996). Sympathetic control of skeletal muscle function: possible co-operation between noradrenaline and neuropeptide Y in rabbit jaw muscles. Neurosci Lett.

[29] Celichowski J, Mrówczyński W, Krutki P, Górska T, Majczyński H, Sławińska U (2006). Changes in contractile properties of motor units of the rat medial gastrocnemius muscle after spinal cord transection. Exp Physiol.

[30] Häbler HJ, Jänig W, Krummel M, Peters OA (1994). Reflex patterns in postganglionic neurons supplying skin and skeletal muscle of the rat hindlimb. J Neurophysiol.

[31] Vissing SF (1997). Differential activation of sympathetic discharge to skin and skeletal muscle in humans. Acta Physiol Scand Suppl.

[32] Hill JM, Kaufman MP (1998). Central command, but not muscle reflex, stimulates cutaneous sympathetic efferents of cats. Am J Physiol.

[33] Lee TK, Lois JH, Troupe JH, Wilson TD, Yates BJ (2007). Transneuronal tracing of neural pathways that regulate hindlimb muscle blood flow. Am J Physiol Regul Integr Comp Physiol.

[34] Dampney RAL, McAllen RM (1988). Differential control of sympathetic fibres supplying hindlimb skin and muscle by subretrofacial neurones in the cat. J Physiol.

[35] Kaufman MP, Longhurst JC, Rybicki KJ, Wallach JH, Mitchell JH (1983). Effects of static muscular contraction on impulse activity of groups III and IV afferents in cats. J Appl Physiol Respir Environ Exerc Physiol.

[36] Mense S, Stahnke M (1983). Responses in muscle afferent fibres of slow conduction velocity to contractions and ischaemia in the cat. J Physiol.

[37] Adreani CM, Hill JM, Kaufman MP (1997). Responses of group III and IV muscle afferents to dynamic exercise. J Appl Physiol.

