# Supplementary figures and images for: Maintenance of contractile force of the hind limb muscles by the somato-lumbar sympathetic reflexes
Source: J Physiol Sci. 2021 May 21;71:15. doi: 10.1186/s12576-021-00799-w (PMC10717212; doi:10.1186/s12576-021-00799-w)

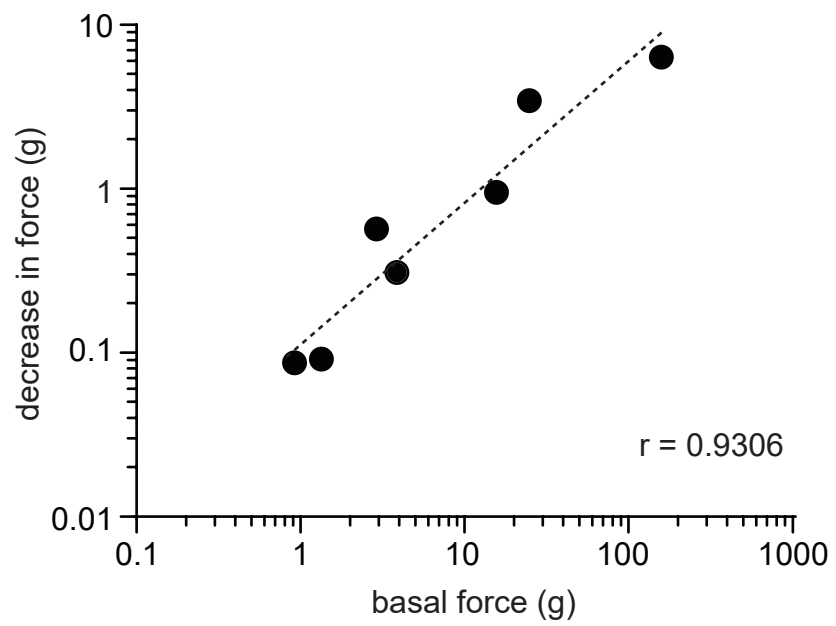

Additional file 1

Supplement: Supplementary file 1 — Additional file 1. Correlation between basal force amplitudes and decrease in force amplitudes following transection of the ipsilateral LST. In the present method, the triceps surae muscles were minimally stretched, so that the basal force recorded (ranging between 0.9 g and 24 g in individual rats) was much less than that of the maximum force. This graph included an additional data, in which the nearly maximum force (160 g) was recorded by using modified methods as described below. The left hind limb was firmly fixed by using bone clamps (STS-A, Narishige, Tokyo, Japan), the triceps surae muscles were stretched with a weight of 50 g and connected to another model of transducer (LVS-1KA, Kyowa Electronics Instruments, 10 N capacity). Strength of correlation was analyzed by Pearson’s method. [file 12576_2021_799_MOESM1_ESM.pdf]

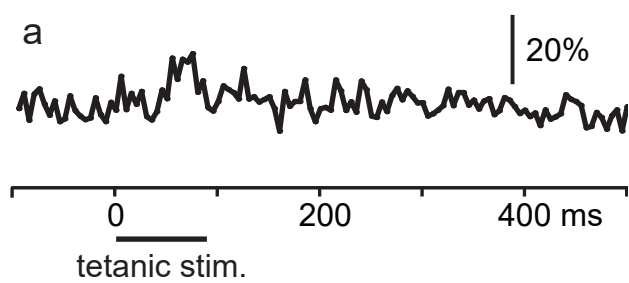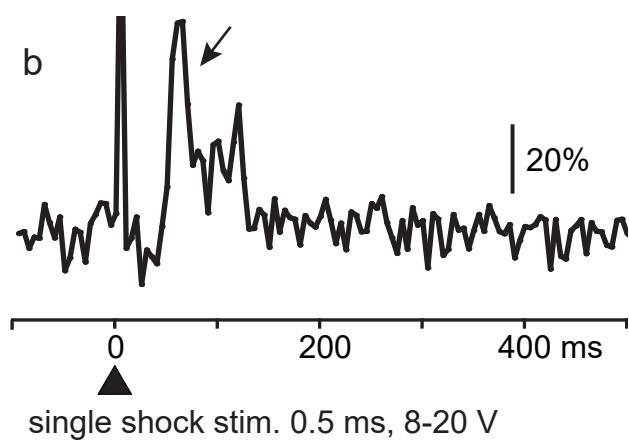

Additional file 2

Supplement: Supplementary file 2 — Additional file 2. Lumbar sympathetic nerve activity in spinalized animals after the administration of muscle relaxants. a: The tetanic stimulation of the intact tibial nerve at 2 T, which caused tetanic contractions in the triceps surae muscles, scarcely induced any reflexive activity in the lumbar sympathetic nerve after muscle relaxation (n = 4 from four rats). b: Reflex discharges induced by single-pulse stimuli with a duration of 0.5 ms delivered every 3 s, with a supramaximal intensity (8–20 V) to excite group III and IV fibers of the tibial nerve after muscle relaxation (n = 3 from three rats). Vertical bars indicate percent changes relative to the prestimulus values. [file 12576_2021_799_MOESM2_ESM.pdf]
